# Origin and behavior of radionuclides in sediment core: a case study of the sediments collected from man-made reservoirs located in the past mining region in Central Slovakia

**DOI:** 10.1007/s11356-019-04136-y

**Published:** 2019-01-16

**Authors:** Katarzyna Szarlowicz, Marcin Stobinski, Ladislav Hamerlik, Peter Bitusik

**Affiliations:** 1 Faculty of Energy and Fuels, AGH University of Science and Technology, al. A. Mickiewicza 30, 30-059 Krakow, Poland; 2 Faculty of Natural Sciences, Matej Bel University, Tajovskeho 40, 97401 Banská Bystrica, Slovakia; 3 Institute of Geological Sciences, Polish Academy of Sciences, Twarda 51/55, 00-818 Warsaw, Poland

**Keywords:** Radionuclides, Sediments, Artificial reservoirs, Gamma spectrometry, Chemometric analysis, Pollution

## Abstract

The analyzed sediments were taken from the man-made reservoirs (Velka Richnava, Rozgrund and Vindsachta) located in an area intensively mined for polymetallic ores since the end of the eleventh century (Banska Stiavnica region, Central Europe). The aims of this study were to determine the radioactivity of natural (^226^Ra, ^228^Th, ^210^Pb) and artificial (^137^Cs and ^241^Am) radionuclides, compare the radionuclides’ distribution, and indicate the correlation of radioisotopes and their origin related to sediment properties. Two analytical techniques were used. ^228^Th, ^226^Ra, ^241^Am, and ^137^Cs were measured by means of gamma spectrometry and ^210^Pb was determined by its daughter radionuclide ^210^Po using alpha spectrometry. The results showed that the highest mean level of ^226^Ra (42.6 Bq·kg^−1^), ^228^Th (49.7 Bq·kg^−1^) and ^210^Pb (75.2 Bq·kg^−1^) was in the sediments collected from Rozgrund. The radioactivity of ^137^Cs and ^241^Am were present at a higher level in the layer related to Chernobyl (1986) accident and nuclear weapon test (1950/1960). The distribution of natural radionuclides was quite similar in all reservoirs. Chemometric analysis confirmed the radionuclides’ origin and correlation between the analyzed parameters.

## Introduction

Radioactivity is a natural part of the environment. Radionuclides are present in every environmental component (air, water, sediments, soils, plants, or animals). The naturally occurring radioisotopes can be divided into three groups, depending on their origin. Primordial radionuclides have existed since the Earth was formed, around 4.6 × 10^9^ years ago, while cosmogenic are produced in nuclear reactions between and the constituents/elements present in the atmosphere or the surface of the earth. The third group contains the radionuclides from the radioactive decay series (uranium–radium (i.e., –^226^Ra–^222^Rn–^218^Po–^214^Pb–^214^Bi–^214^Po–^210^Pb–), uranium–actinium (i.e., –^231^Th–^227^Ac–^223^Ra–^215^Po–^211^Bi–^207^Pb–), and thorium (i.e., –^228^Ra–^228^Ac –^228^Th–^220^Rn–^212^Pb–^212^Bi–^208^Tl–)) (Isaksson and Raaf 2016). All minerals and raw materials contain radionuclides of natural origin. Certain industrial activities (coal industry, metal mining, smelting, mineral sands, etc.) can give rise to significantly enhanced radiation exposures. Material giving rise to these enhanced exposures has become known as naturally occurring radioactive material (NORM). Mining and processing of metal ores may also generate large quantities of NORM waste. In iron smelting, radioactivity can even reach about 100 kBq∙kg^−1^ for ^210^Pb and ^210^Po in dust (Cooper 2003).

However, with the discovery of neutrons by James Chadwick in 1932, a number of radionuclides of non-natural origin began to be created (Keller et al. 2011). Uncontrolled release of artificial radionuclides is a big problem for the environment safety, due to disturbance of the natural background of ionizing radiation. The dominant sources of anthropogenic radioactivity are accidental releases such as Chernobyl (1986) and global weapons testing 1950/1960 (maximum deposits in 1963). In fact, large areas of Europe taking into account all environmental components were contaminated (Hodge et al. 1996; UNSCEAR 2000; Evangeliou et al. 2013). From radio-ecological point of view, the most important elements are the long-lived radionuclides, i.e., ^137^Cs (T_1/2_ = 30.05 y) or ^90^Sr (T_1/2_ = 28.8 y). Nowadays, attention should be also paid to ^241^Am (T1/2 = 432.6 y), the element which is formed exclusively as a result of nuclear fission processes as a daughter nuclide of plutonium ^241^Pu (Appleby et al. 1991; DOE 2000; Lehto and Hou 2011). All natural or artificial radionuclides can be present in sediments. Sediments can be treated as a repository for different kinds of contaminants including radionuclides (Skwarzec 1995; Appleby 2001; Szarlowicz et al. 2018). In this work, we would like to present the level of ^137^Cs, ^241^Am, ^226^Ra, ^210^Pb, and ^228^Th in the sediment samples taken from the man-made reservoirs situated in the Stiavnicke vrchy Mountains in the inner belt of the West Carpathians. The mountain range was formed during several stages of andesitic and rhyolitic volcanism in the Middle and Late Miocene (Chernyshev et al. 2013).

Therefore, the adjacent area around the town Banska Stiavnica is rich with iron and polymetallic ores containing lead, zinc, copper, gold, and silver (Konečný 1971). This region, together with some other sites in the Central Slovakia, occupied a dominant position among the medieval mining regions in Europe. History of mining in Banska Stiavnica surroundings has been recorded since the end of the eleventh century to the turn of the twentieth century, although traces of the mining have been documented from the Bronze Age (Labuda 2016; Lichner 2002).

The surveyed reservoirs have been a part of the hydro-energetic system that was completed during the biggest expansion of mining between the beginning of the eighteenth and the first half of the nineteenth century. Water from this system provided energy for operating of mining machines, ore cleaning facilities, and smelting works (Novák 1977). The reservoirs and other technical monuments situated in a unique, man-modified landscape were inscribed in the World Cultural and Natural Heritage List UNESCO in 1993.

The previous multi-proxy palaeolimnological studies (Bitušík et al. 2018) have documented a potential of the sediments for the reconstructions of an ontogeny of these reservoirs and for expanding the current knowledge of changes in mining landscapes that are rarely described in historical documents.

The main focus of this study was to compare distribution, correlation of radioisotopes, and their origin related to sediments properties for three reservoirs Velka Richnava, Rozgrund, and Vindsachta. To do this, basic chemometric tools were used.

## Material and method

### Study sites

The surveyed reservoirs are located in an open landscape with mosaic of mixed forests, grasslands, and settlements. While Velka Richnava (RICH) and Vindsachta (VIND) belong to the same reservoir group and were interconnected via gallery, Rozgrund (ROZ) is about 20 km away. The bedrock of VIND and RICH and their surroundings consists of pyroxene and amphibole-pyroxene andesite with accessory biotite, quartz, and garnet altered by successive hydrothermal activity. The surroundings of ROZ are more heterogeneous and consist of mineralogical varieties of andesite and diorite and hydrothermally altered veins. Deluvial deposits, mostly loamy stony and stony screes, cover the bedrock of all reservoirs (Konečný et al. 1998).

While ROZ has served as a source of drinking water with protected watershed since the second half of the 1920s, the surroundings of RICH and VIND are partially urbanized. The function of both reservoirs is primarily to support recreation, such as swimming, boating, and angling. A more detailed description of the studied sites is given in Table 1.

**Table 1:** Characteristics of the surveyed reservoirs

Reservoir	Velka Richnava	Vindsachta	Rozgrund
Coordinates	N 48°25′37″ E 18°50′46″	N 48°26′03″ E 18°51′22″	N 48°28′39″ E 18°52′32″
Year of building	1740	1715	1743
Altitude [m a.s.l.]	725	688	703
Area [m^2^]	82,830	46,540	54,350
Max depth [m]	19.5	12.7	20.9
Volume [m^3^]	~ 666,000	~ 285,000	~ 575,000

### Sampling

The sediment core samples were collected using the UWITEC Niederreiter 60 (Ø 6.0 cm) hydraulic coring system. From each reservoir, two sediment cores were obtained (core length RICH—1.84, 1.10 m; ROZ—4.20, 3.53 m; and VIND—1.2, 1.03 m). The core samples were divided into 1-cm sections. They were packed into plastic bags and stored in a refrigerator for later analysis. Only chosen samples from the longer core were analyzed.

### Preparation and measurements

The samples were dried in a room temperature, grinded in a mortar, and packed into the calibrated flat round vessels (volume 1.5 cm^3^, 2.7 cm internal diameter). The radioisotope determination of the samples was carried out by two different techniques. For gamma radionuclides, high-resolution gamma spectrometry with HPGe detector (Canberra model BE3830 with carbon composite window and relative efficiency 34%) was used. The radioactivity of ^137^Cs and ^241^Am was found by photo peaks at 661.6 keV and 59.5 keV respectively. The photo peaks used for ^226^Ra came from ^214^Pb (351.9 keV, 295.2 keV) and ^214^Bi (1764.5 keV, 1120.3 keV, 609.3 keV). The ^228^Th was determined by gamma lines from ^212^Pb (238.6 keV), ^212^Bi (727.3 keV), and ^208^Tl (2614.5 keV, 583.1 keV).

^210^Pb radioactivity was determined by means of its daughter alpha radionuclide ^210^Po. Based on the amount of the taken sediment, the alpha spectrometry is the most sensitive measurement method. The radiochemical procedure consisted of microwave sample (0.2 g) digestion with concentrated nitric and hydrochloric acids (Anton Paar Multiwave Pro), evaporation to dryness and dissolving of the residue in 2 mol dm^−3^ HCl, and the source preparation in the presence of hydroxylamine hydrochloride and sodium tri-citrate. ^208^Po was added to every sample in order to calculate the efficiency of the procedure. ^210^Pb was calculated after two depositions of polonium in time of about 6 months (Szarlowicz et al. 2013). Sources were measured 3–5 days. An Alpha spectrometer model 7401 was used. All spectra were analyzed using Genie—2000 software. In both cases, to check the method, the reference materials from IAEA (International Atomic Energy Agency, IAEA-447, IAEA-RGU-1, IAEA-RGTh-1) were measured. In alpha spectrometry, the blank solution also was measured. As for gamma, the radionuclide background was checked monthly.

### Organic matter content

The organic matter content was determined as loss-on-ignition (LOI) and expressed as the percentage weight loss after combustion at 550 °C for 4 h (Heiri et al. 2001).

### Statistical data analysis

To distinguish relevant information out of the obtained data, the data matrix consisting of all analyzed features of all samples was analyzed statistically (separately for each reservoir). Chemometric tools, i.e., cluster analysis (CA) and principal components analysis (PCA), were used (using Statistica 10 and Stagraphics Centurion software).

## Results and discussion

The activity of the natural radionuclide and other parameters are shown in Table 2. The uncertainty of the radioactivity measurements was not higher than 20%.

**Table 2:** Natural radionuclides’ concentration and basic data for the samples from the studied reservoirs

	VIND (*n* = 13)			RICH (*n* = 13)			ROZ (*n* = 10)		
	Average	Minimum	Maximum	Average	Minimum	Maximum	Average	Minimum	Maximum
^228^Th [Bq·kg^−1^]	38.9	29.0	64.5	35.5	27.1	50.4	49.7	40.2	68.1
^226^Ra [Bq·kg^−1^]	37.8	27.4	55.5	33.5	26.8	44.4	42.6	28.5	68.5
^210^Pb [Bq·kg^−1^]	62.1	21.0	226.0	35.5	15.3	251.0	75.2	29.1	278.0
Organic matter [%]	10.5	7.4	15.2	7.1	3.8	12.4	9.5	7.1	13.3
Density [g cm^−3^]	1.1	0.8	1.3	0.9	0.5	1.3	0.7	0.5	1.0
Depth [cm]	45	2	103	86	1	171	136	3	321

It was found that the levels of average radioactivity of ^226^Ra and ^228^Th in the RICH and VIND reservoirs were similar. In ROZ, the highest radioactivity of ^226^Ra was observed. The same situation was applied to the levels of ^228^Th with the highest average level for ROZ. The distribution of ^226^Ra and ^228^Th within the sediment core showed a very similar pattern in all three lakes. The distribution was rather regular, only minor deviations were observed. The levels of radioactivity were connected with mineralogical composition of the sediments as coarse grained sand and gravel in the older layers were replaced by sand, silt, and clay in the younger ones (Bitušík et al. 2018). So, the activity concentration can show local deviations from that of ^238^U or ^232^Th (mother radionuclide) due to weathering and other environmental factors.

The results of ^210^Pb radioactivity for all three lakes are also presented in Table 2. The ^210^Pb radioactivity in the top layers was almost on the same level, going deeper the values decreased exponentially (see Chamutiova et.al 2018). The tendency of the lead radioactivity changes in RICH and ROZ indicate similarity of the sediments’ deposition processes. Besides, there were also visible some small irregularities along the sediment’s core. A little different situation was observed for VIND; here, a regular decrease of radioactivity was observed. The obtained results were used to determine the age of sediments using the ^210^Pb method. In the RICH, the 171-cm long sediment core was deposited over the last ca. 174 years. The sediment core from ROZ represents the sediments from the last 191 years. The 82 cm in the VIND started to deposit at the beginning of the nineteenth century. The ^210^Pb and ^137^Cs geochronology results have been published (Bitušík et al. 2018, Chamutiová et al. 2018).

Artificial radionuclides were also identified. In some layers, it was possible to perform quantitative determination but in the other, only qualitative information was obtained. In some of the samples (in the majority of ROZ), the ^137^Cs radioactivity was below the limit of detection. In ROZ, the ^137^Cs was only determined in the uppermost layer. The value was about 128.0 ± 4.5 Bq·kg^−1^. The ^137^Cs radioactivity in the VIND reservoir was present in the 26 cm of the core in the range from 41.5 ± 3.8 to 202.0 ± 6.3 Bq·kg^−1^. Regarding ^137^Cs radioactivity in RICH, it was identified in the samples 0–1 cm (540 ± 7 Bq·kg^−1^), 10–11 cm (11 ± 3 Bq·kg^−1^), 40–41, and 50–51 cm (< 6 Bq·kg^−1^).

In the VIND reservoir, ^241^Am was well defined. The presence of ^241^Am was about 5.4 ± 1.2 Bq·kg^−1^ and it was observed from 1 to 32 cm. The highest level of ^241^Am was found in the 20–21 cm (1965) and 31–32 cm (1953) layers. In RICH analyzed spectra, only counts from americium at 50–51 cm (1956) were observed, but the number of counts was too small to quantify them. Regarding ROZ, there were also some counts dedicated to the uppermost layer and also to 110–111 (1962). The year in brackets represents the year ascribed to the layer determined by ^210^Pb dating.

**Fig. 1:**
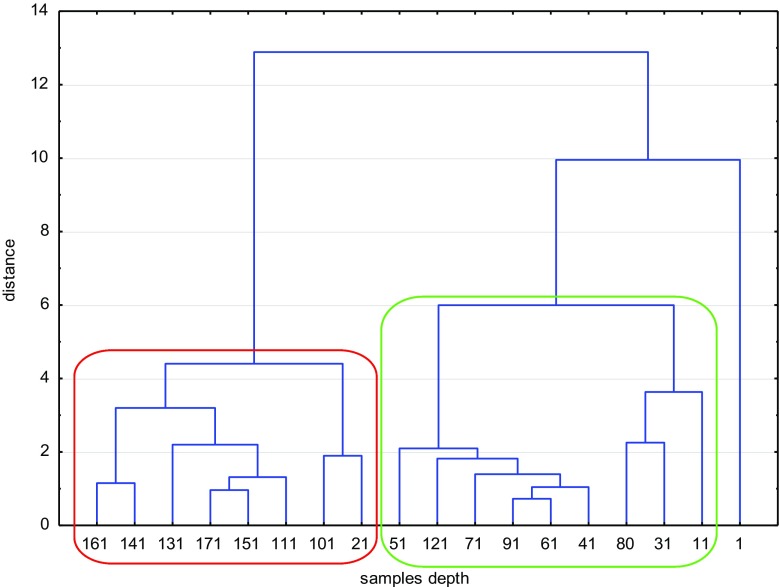
Cluster analysis for the RICH lake. Dendrogram aglomerated with Ward’s method, Euclidean distance

Statistical correlation analysis was performed in order to evaluate the factors having influence on the radionuclides concentrations. In all three reservoirs, the correlation showed a very strong correlation between the analyzed parameters. The correlation coefficient according to Spearman rank-order was done. The best correlation was found in VIND. The values of the correlation coefficients are presented in Table 3. A positive correlation of ^137^Cs with ^241^Am, ^210^Pb, and organic matter was observed and a negative one with depth. ^137^Cs and ^241^Am have the same anthropological origin. They were present only in the younger layers. To compare the layers, the graphical presentation of CA with Ward’s method and Euclidean distance was done separately for each reservoir. All results were standardized prior to chemometric analysis. Figure 1 represents the results for RICH.

**Table 3:** Spearman’s rank-order correlation for the VIND lake. Marked correlation coefficients are significant with *p* < 0.50

	Organic matter	Density	^137^Cs	^228^Th	^226^Ra	^241^Am	^210^Pb
Depth	− 0.857	0.989	− 0.839	− 0.140	− 0.187	− 0.794	− 0.990
Organic matter		− 0.857	0.748	0.179	0.024	0.686	0.819
Density			− 0.830	− 0.096	− 0.140	− 0.806	− 0.976
^137^Cs				0.091	0.343	0.701	0.807
^228^Th					0.564	0.080	0.135
^226^Ra						0.179	0.150
^241^Am							0.768

Evidently, the first layer was totally different from the others. Considering the remaining layers, two main clusters could be distinguished. The first cluster (green line) represented in the majority of the samples at the depth above 100 cm (up to 1933), while the second (red line) contained the shallower layers (older samples up to ca 1840). The samples 21 and 121 were the exceptions. It can result from the sediments’ composition. Very-low level of organic matter content in the sample 21 can be correlated with high mineralization as a result of oxygen presence caused by drainage of the reservoir. On contrary, the sample 121 was found in the cluster with high content of organic matter caused by erosive contact (Bitušík et al. 2018).

**Fig. 2:**
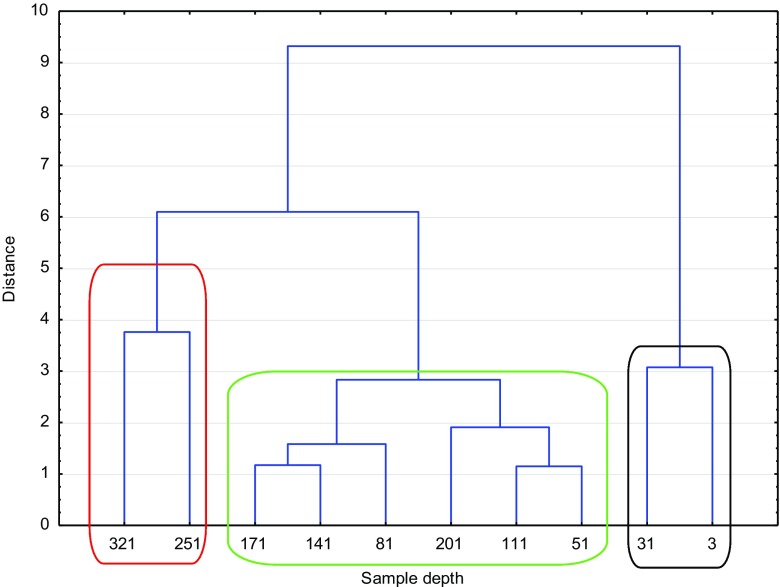
Cluster analysis for the ROZ lake. Dendrogram aglomerated with Ward’s method, Euclidean distance

**Fig. 3:**
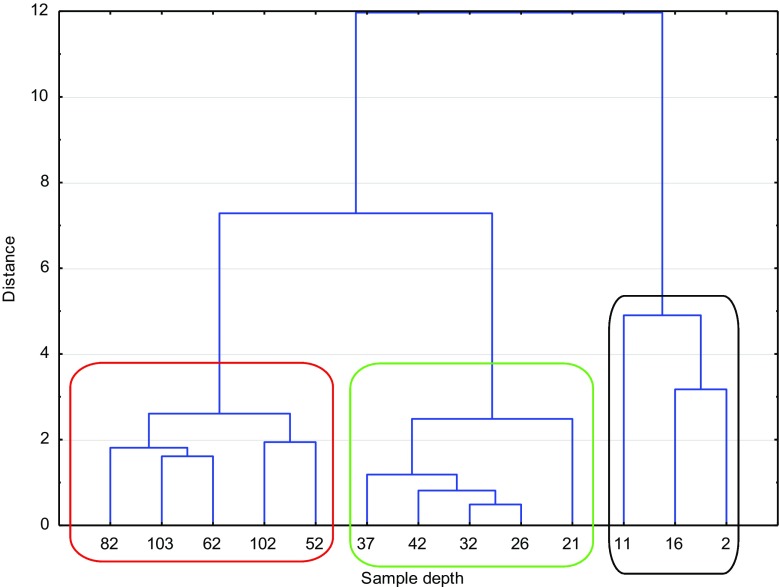
Cluster analysis for the VIND lake. Dendrogram aglomerated with Ward’s method, Euclidean distance

In Figs. 2 and 3, the dendrograms for ROZ and VIND are presented. Here, three main clusters could be distinguished. In both reservoirs, the first cluster (black line) represented the youngest samples (ROZ 2013–1996 and VIND 2013–1987) and these were sediments with characteristic totally different from the others. In the cluster marked with green, sediments formed in the years 1936–1973 for VIND and 1940–1973 for ROZ are gathered. The third cluster (red line) consists of the oldest sediments layers (up to 1816—VIND and 1825—ROZ, respectively).

**Fig. 4:**
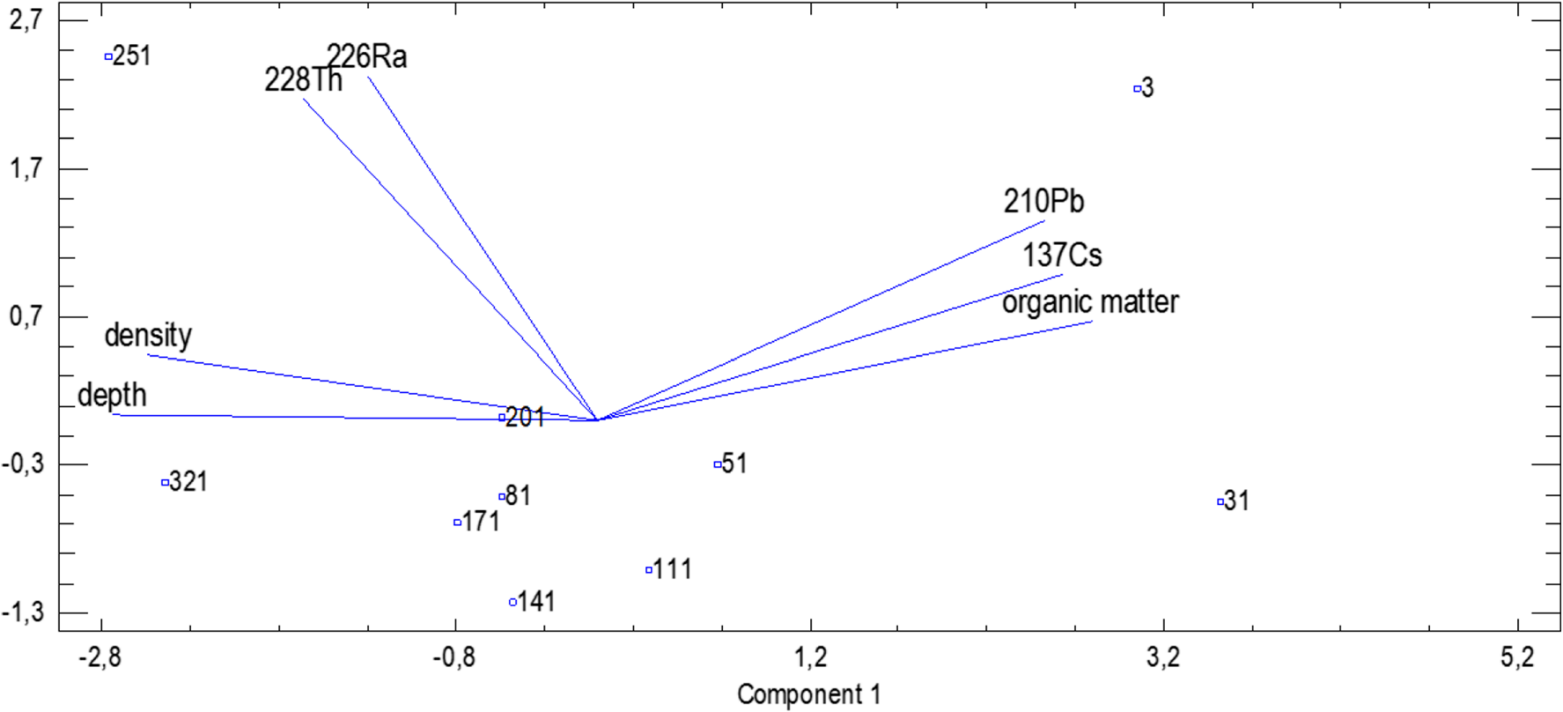
The biplot with projection of the variables and cases onto the plane of the first two principal components for the ROZ lake

**Fig. 5:**
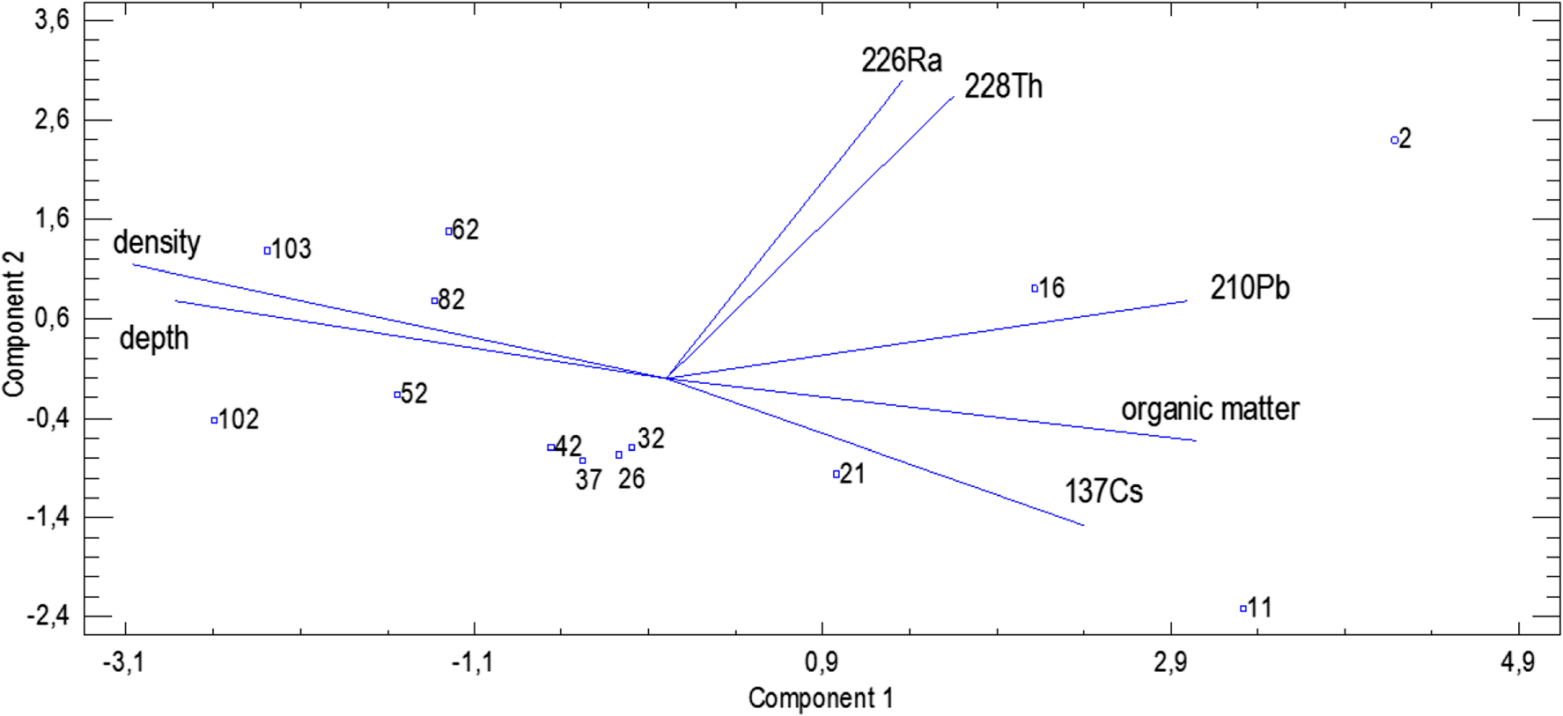
The biplot with projection of the variables and cases onto the plane of the first two principal components for the VIND lake

Regarding the origin of the radionuclides, the PCA makes the situation quite clear. Based on the PCA analysis with Kaisser’s criterion, it was found that 82.8% (ROZ) and 85.9% (VIND) of the variability contained in the considered set of data can be described using the two principal components. In Figs. 4 and 5, it is shown that the first principal component was positively correlated with depth and density. Negative correlation was ascribed to the organic matter content, ^137^Cs and ^210^Pb. ^228^Th and ^226^Ra were described by the second principal component.

**Fig. 6:**
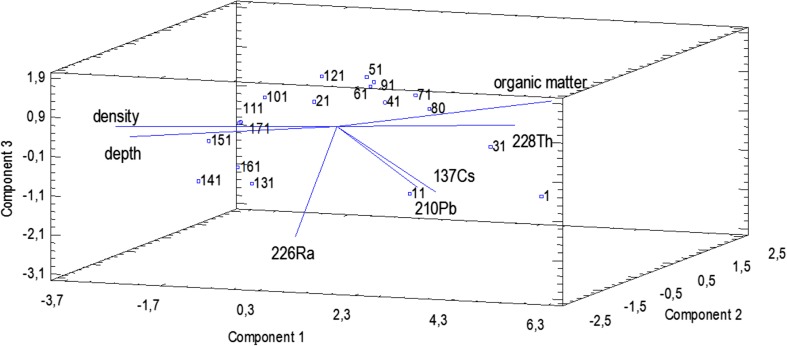
The biplot with projection of the variables and cases onto the plane of the first three principal components for the RICH lake

In RICH, three principal components were needed for the description of the component relationship in the sediments (Fig. 6). They represent 82.5% of the variance.

Additionally, factor analysis (PCA factoring) with varimax rotation was used. In Table 4, the factor loadings matrix after varimax rotation is presented. Vari factor-1 was positively correlated with depth and density and negatively with the organic matter content and ^228^Th. ^137^Cs and ^210^Pb were negatively correlated with vari factor-2, and vari factor-3 represents ^226^Ra.

**Table 4:** Factor loading matrix after varimax rotation for the RICH lake

	Vari factor—1	Vari factor—2	Vari factor—3
Depth	0.758	0.317	0.122
Organic matter	− 0.764	− 0.388	− 0.310
Density	0.814	0.356	0.049
^137^Cs	− 0.173	− 0.970	− 0.091
^228^Th	− 0.710	0.008	0.153
^226^Ra	0.079	0.064	0.978
^210^Pb	− 0.255	− 0.958	− 0.025

The Principal Component analysis revealed that the activities of ^137^Cs and ^210^Pb, as well as the concentration of organic matter in the sediments cores, are strongly and positively correlated, simultaneously being negatively correlated with the profile depth and sediments’ density. It can be attributed to the fact that both anthropogenic ^137^Cs and natural ^210^Pb (mainly its unsupported component) were delivered to the reservoir area in the result of dry and wet deposition (long distance character). It can also be observed in the analysis of the correlation matrix (Table 3). Those radionuclides reveal strong affinity to organic matter, so the higher content of organic matter, the higher activity of radionuclides (Bolsunovsky et al. 2005; Szarłowicz et al. 2011, Stobinski et al. 2014). Going deeper into the profile, the content of organic matter decreases which is accompanied by the increase of mineral content and density of the examined sediments. ^228^Th and ^226^Ra (natural radionuclides) are formed in situ being the component of mother rock, soil, or particulate matter and were constantly delivered from the surrounding area to the reservoirs (local character).

## Conclusions

For the first time, in respect to the radionuclides in the selected reservoirs, such research was proposed, performed, and discussed. The following can be concluded:

1. the radioactivity levels of ^226^Ra and ^228^Th are comparable to those present in the Earth’s crust and in the sediments taken from stream and river sediments in Central Slovakia region (UNSCEAR 2000; Cabáneková and Melicherová 2015);
2. the distribution of ^210^Pb can explain the process of sediment’s deposition;
3. in none of the sediments’ cores, there was no distinct ^137^Cs maximum peak determined. However, relating to the time scale (based on ^210^Pb dating), elevated levels of the radionuclide were found;
4. it was confirmed that ^241^Am is also a good time marker, the presence of maximum in sediment core verified it. It can be used as an additional confirmation of the date especially in relation to nuclear weapon test;
5. despite the different location and functions of the reservoirs (RICH and VIND—recreation, ROZ—a drinking water reservoir), the sediments contain radionuclides at a quite similar level. Small differences are mainly related to the distribution of the artificial radionuclides and composition of the sediments;
6. it is assumed that both ^137^Cs and ^241^Am were at the measurable level in VIND due to the highest content of organic matter. Besides, it suggests that the function of the reservoir has no influence on radionuclides distribution;
7. Summarizing, radiochemical analysis of the sediments together with statistical approach provides valuable information about the origin and behavior of radionuclides in the environment. Chemometric analysis confirmed the radionuclides origin and correlation between the parameters. The presented chemometric analysis suggests that the applied methodology might be useful in the analyses of the date deposition of the layers in other reservoirs or lakes, respectively in the region.
